# Social epidemiology of Fitbit daily steps in early adolescence

**DOI:** 10.1038/s41390-023-02700-4

**Published:** 2023-06-23

**Authors:** Jason M. Nagata, Sana Alsamman, Natalia Smith, Jiayue Yu, Kyle T. Ganson, Erin E. Dooley, David Wing, Fiona C. Baker, Kelley Pettee Gabriel

**Affiliations:** 1 Department of Pediatrics, University of California, San Francisco, CA USA; 2 Factor-Inwentash Faculty of Social Work, University of Toronto, Toronto, ON Canada; 3 Department of Epidemiology, University of Alabama at Birmingham, Birmingham, AL USA; 4 Exercise and Physical Activity Resource Center, University of California, San Diego, CA USA; 5 Center for Wireless and Population Health Systems, University of California, San Diego, La Jolla, CA USA; 6 Herbert Wertheim School of Public Health and Human Longevity Science, La Jolla University of California, San Diego, La Jolla, CA USA; 7 Center for Health Sciences, SRI International, Menlo Park, CA USA; 8 School of Physiology, University of the Witwatersrand, Johannesburg, South Africa

## Abstract

**Background:**

Sociodemographic disparities in adolescent physical activity have been documented but mostly rely on self-reported data. Our objective was to examine differences in device-based step metrics, including daily step count (steps d^−1^), by sociodemographic factors among a diverse sample of 10-to-14-year-old adolescents in the US.

**Methods:**

We analyzed prospective cohort data from Year 2 (2018–2020) of the Adolescent Brain Cognitive Development (ABCD) Study (*N* = 6460). Mixed-effects models were conducted to estimate associations of sociodemographic factors (sex, sexual orientation, race/ethnicity, household income, parental education, and parental marital status) with repeated measures of steps d^−1^ over the course of 21 days.

**Results:**

Participants (49.6% female, 39.0% racial/ethnic minority) accumulated an average of 9095.8 steps d^−1^. In mixed-effects models, 1543.6 more steps d^−1^ were recorded for male versus female sex, Black versus White race (328.8 more steps d^−1^), heterosexual versus sexual minority sexual orientation (676.4 more steps d^−1^), >$200,000 versus <$25,000 household income (1003.3 more steps d^−1^), and having married/partnered parents versus unmarried/unpartnered parents (326.3 more steps d^−1^). We found effect modification by household income for Black adolescents and by sex for Asian adolescents.

**Conclusions:**

Given sociodemographic differences in adolescent steps d^−1^, physical activity guidelines should focus on key populations and adopt strategies optimized for adolescents from diverse backgrounds.

**Impact:**

- Sociodemographic disparities in physical activity have been documented but mostly rely on self-reported data, which can be limited by reporting and prevarication bias.
- In this demographically diverse sample of 10–14-year-old early adolescents in the U.S., we found notable and nuanced sociodemographic disparities in Fitbit steps per day.
- More daily steps were recorded for male versus female sex, Black versus White race, heterosexual versus sexual minority, >$100,000 versus <$25,000 household income, and having married/partnered versus unmarried/unpartnered parents.
- We found effect modification by household income for Black adolescents and by sex for Asian adolescents.

## Introduction

While the psychological and physical benefits of physical activity have been thoroughly demonstrated in the literature, less than one quarter of United States (U.S.) adolescents meet the physical activity guidelines recommended by the U.S. Department of Health and Human Services (60 min per day, 7 days per week).^1^ Furthermore, recent studies relying on self-reported physical activity suggest that with the onset of the coronavirus disease 2019 (COVID-19) pandemic, the proportion of adolescents meeting these guidelines has fallen from 16% to 9%, with marked differences across racial/ethnic groups and socioeconomic backgrounds.^2^

Self-report measures of physical activity are limited by reporting and prevarication biases. Recent studies have validated the physical activity measures of wrist-worn activity monitors, such as Fitbit devices. Fitbit devices can provide accurate and consistent measurements, including step count, in adolescents over long periods of time.^3,4^ Among the various physical activity measures on Fitbits, previous studies have demonstrated daily step count as a valid and easily accessible measure to monitor physical activity.^5^ Step count offers a more accurate measure in diverse populations as it does not rely on photoplethysmography (PPG) derived measures of heart rate, which have issues with validity specifically in individuals with darker skin tones.^6^ Step count, like other measures of physical activity, is inversely related to the risk of mortality and cardiovascular disease (CVD) morbidity.^5,7^ Studies have shown that every additional 1000 steps per day (steps d^−1^) are protective against CVD morbidity and mortality in adults.^5^ While some studies have described physical activity data from wrist-worn research-grade accelerometers like GENEActiv or ActiGraph, these studies have been limited to relatively small samples of adolescents for short periods of time, typically 7 days or fewer.^8^ In contrast, the self-charge feature of consumer-based wearables like Fitbit makes them more practical for long-term data collection since research-grade devices have a limited battery life, and charging cables are typically not provided to participants. Few studies have examined daily step patterns over extended periods of time in large, racially and ethnically diverse community-dwelling samples of U.S. adolescents. With the availability of Fitbit wearables, this research is now feasible.^9^

Social epidemiology is a field that focuses on the intersection of structural and social factors on health, informed by biopsychosocial theory or how biological, psychological, and social factors can impact health.^10–12^ For instance, demographic (e.g., sex, race/ethnicity, and sexual orientation) and socioeconomic (e.g., income and education) factors can impact an individual’s health outcomes. Sociodemographic disparities in physical activity have been documented but mostly rely on self-reported data. For instance, a 2018 study surveying over 9000 adolescents found that females were less physically active than their male counterparts.^13^ Racial/ethnic minorities and low-income populations also reported less physical activity.^13^ Furthermore, studies have identified that sexual minority adolescents are less likely to be physically active.^14,15^ However, there is a paucity of studies examining disparities in device-based measures of physical activity collected over extended periods. Considering how crucial exercise and physical activity are for adolescent well-being, it is valuable to better understand these disparities using device-based methods.

Given these gaps, the objective of our study was to analyze daily step patterns among a socio-demographically diverse, population-based sample of 10-to-14-year-old children in the U.S. Building on previous studies using self-reported physical activity,^13–16^ we hypothesized that differences in daily step patterns would exist by race/ethnicity, sex, sexual orientation, and socioeconomic status. Specifically, we hypothesized that racial/ethnic minority, female, sexual minority, and low-income adolescents would have lower daily steps than their peers.

## Methods

Cross-sectional data from Year 2 (2018–2020) of the Adolescent Brain Cognitive Development (ABCD) Study (4.0 release; 2018–2020, ages 10–14 years) were analyzed. The ABCD Study is the largest long-term longitudinal study of brain development and health in the U.S. The study sample, recruitment, procedures, and measures have been previously described.^17^ Data analyzed in this study were collected by Fitbit Charge 2 devices at Year 2 of the ABCD assessment. Participants with missing data for the steps d^−1^ variables were excluded, leaving a total sample of 6460 adolescents whose demographic and physical activity data are detailed in Table 1. Centralized institutional review board (IRB) approval was received from the University of California, San Diego (UCSD). The University of California, San Francisco (UCSF) approved the secondary data analyses. Study sites obtained approval from their local IRBs. Caregivers provided written informed consent, and each child provided written assent.

**Table 1:** Sociodemographic and physical activity characteristics of Adolescent Brain Cognitive Development (ABCD) Study participants (*N* = 6460).

Sociodemographic characteristics (baseline)	Mean (SD)
Age (years)	12.0 (0.7)
Sex (%)	
Female	49.6
Male	50.4
Race/ethnicity (%)	
White	61.0
Latino/Hispanic	17.2
Black	12.2
Asian	5.2
Native American	3.2
Other	1.3
Sexual minority (%)	
No	88.4
Yes	3.9
Maybe	3.6
Don’t understand the question	3.1
Decline to answer	0.9
Household income (%)	
≤$24,999	12.0
$25,000–$49,999	19.2
$50,000–$74,999	18.9
$75,000–$99,999	15.6
$100,000–$199,999	26.0
≥$200,000	8.2
Parent education, average years	16.5 (2.5)
Parent marital status (%)	
Parent married/partnered	73.8
Parent not married/unpartnered	26.2
Data collection period (%)	
Pre-COVID	83.3
During COVID	16.7
Physical activity variables (baseline, weekday, weekend)	
Total steps per day	9101.7 (3203.8)
Total steps per day on weekdays	9441.9 (3404.6)
Total steps per day on weekends	8121.849 (3714.1)

ABCD propensity weights were applied based on the American Community Survey from the US Census.

*SD* standard deviation.

## Measures

### Independent variables

Sex (female or male), race/ethnicity (White, Latinx/Hispanic, Black, Asian, Native American, other), sexual orientation, household income, highest parent education, and parent marital status were self-reported by adolescents and/or their parents. These variables have previously been shown to be associated with self-reported physical activity.^13^

For sexual orientation, participants were asked, “Are you gay or bisexual?”^18^ Response options included: yes, maybe, no, don’t understand the question, or decline to answer. For household income, parents were asked, “What is your total combined family income for the past 12 months? This should include income (before taxes and deductions) from all sources, wages, rent from properties, social security, disability and/or veteran’s benefits, unemployment benefits, workman’s compensation, help from relatives (include child payments and alimony), and so on.” Household income was categorized into ≤$24,999, $25,000–$49,999, $50,000–$74,999, $75,000–$99,999, $100,000–$199,999, or ≥$200,000. When testing for effect modification, household income was dichotomized into less than $75,000 and $75,000 and greater, as this approximated the median household income in the U.S.^19^ For education, parents were asked, “What is the highest grade or level of school you have completed or the highest degree you have received?” and “What is the highest grade or level of school your partner completed or highest degree they received?” Years of parent education were calculated as the average number of years of education for the parent and their partner (if there was a partner) or for the single-parent participant if no partner information was provided. For parent marital status, parents were asked, “Are you now married, widowed, divorced, separated, never married, or living with a partner?” This variable was dichotomized as married/living with a partner or not married/not living with a partner (widowed, divorced, separated, never married).

Given that the COVID-19 pandemic occurred during the data collection period (November 2018-November 2020), we created a COVID variable based on the dates of Fitbit device data collection. “Pre-COVID” was defined as all steps d^−1^ occurring before 3/13/2020 while “During COVID” was defined as all steps d^−1^ occurring on or after 3/13/2020.

### Dependent variable: steps per day

Steps d^−1^ were collected via Fitbit Charge 2 devices (Fitbit Inc., San Francisco, CA) over a 3-week period (21 days). Others have shown that Fitbit devices provide a valid and reliable measurement of daily step count as an estimate of accumulated physical activity in adolescents over long periods of time.^3,20^ We followed best practices to extract, filter, and process data established by the ABCD Study.^3,20^ We included all days with >599 daily minutes of waking wear time within each participant’s 3-week study protocol, collected between November 2018 to November 2020, and a minimum of 1000 steps d^−1^ similar to previous studies.^21–24^

### Statistical analyses

Data analysis was performed in 2022 using Stata 15.1 (StataCorp). Descriptive and mixed-effects models with random effects for participants were used to estimate associations of sociodemographic factors (age, sex, race/ethnicity, sexual orientation, household income, parental education, parent marital status) with repeated measures of steps d^−1^ on (1) all days of the week, (2) weekdays, and (3) weekends, adjusting for site. Repeated measures of daily steps across the 21-day Fitbit protocol were analyzed as the outcome. We expected variation in the association between income and our outcomes by race/ethnicity based on previous literature demonstrating Minorities’ Diminished Returns (MDRs), which describes the persistent disparities in the health of Black people across all domains.^25^ In exploratory analyses, we tested for effect modification by household income in the association between race/ethnicity and steps d^−1^ (e.g., income × race/ethnicity interaction term), as well as effect modification by race/ethnicity in the association between sex and steps d^−1^ (i.e., race/ethnicity × sex interaction terms). Interactions were considered significant with a *p* value of <0.05. Propensity weights were applied to match key sociodemographic variables in the ABCD Study to the American Community Survey from the U.S. Census.^26^

## Results

Table 1 describes the sociodemographic characteristics of the 6460 participants included in this study. The analytic sample was approximately equally distributed by sex (49.6% female) and was diverse by race/ethnicity (39.0% racial/ethnic minority). On average, adolescents accumulated a total mean of 9101.7 (95% confidence interval (CI), 9015.9; 9187.4) steps d^−1^ over the course of 21 days. A total mean of 9441.9 (95% CI, 9350.7; 9533.1) steps d^−1^ were accumulated on weekdays and 8121.849 (95% CI, 8020.8; 8222.9) steps d^−1^ on weekends.

Table 2 shows mixed-effects models examining sociodemographic correlations with steps d^−1^. On average, female adolescents took 1543.6 (95% CI, 1356.7; 1730.5) fewer mean steps d^−1^ than male adolescents (*p* < 0.001). Participants who identified as a sexual minority (responding “yes” to the gay or bisexual question) took 676.4 (95% CI, 129.2; 1223.6) fewer mean steps d^−1^ than their heterosexual peers did. Responding “maybe” to the gay or bisexual question was associated with 958.3 (506.3; 1410.3) fewer mean steps d^−1^ compared to heterosexual adolescents. On average, Black adolescents took 328.8 (95% CI, 39.0; 618.6) more mean steps d^−1^ compared to White adolescents (*p* = 0.028). On weekends, Native American adolescents reported 385.6 (95% CI, 1.2; 770.1) fewer mean steps d^−1^ compared to White adolescents (*p* < 0.049). Each additional year of life among the early adolescent (10–14 years) sample was associated with 282.0 (95% CI, 158.0; 406.0) fewer mean steps d^−1^ (*p* < 0.001). Household income of >$200,000 was associated with 1003.3 (95% CI, 630.3; 1376.3) fewer mean steps d^−1^ when compared to household income <$25,000 (*p* < 0.001). Having single/not married/unpartnered parents was associated with 326.3 (95% CI, 128.0; 524.6) fewer steps d^−1^ compared to adolescents having married/partnered parents (*p* = 0.003). Regarding COVID-19, pandemic days were associated with 2133.5 (95% CI, 1725.7; 2541.2) fewer steps d^−1^ than pre-pandemic days for all adolescents (*p* < 0.001).

**Table 2:** Sociodemographic associations with steps per day in the Adolescent Brain Cognitive Development (ABCD) Study.

Sociodemographic characteristics	Total steps per day		Total steps per day on weekdays		Total steps per day on weekends	
	*B* (95% CI)	*p*	*B* (95% CI)	*p*	*B* (95% CI)	*p*
Age (years)	**−282.0 (−406.0; −158.0)**	**<0.001**	**−266.0 (−394.4; −137.7)**	**<0.001**	**−331.0 (−455.0; −206.9)**	**<0.001**
Sex						
Female	Reference		Reference		Reference	
Male	**1543.6 (1356.7; 1730.5)**	**<0.001**	**1631.6 (1445.2; 1818.0)**	**<0.001**	**1305.4 (1072.0; 1538.9)**	**<0.001**
Race/ethnicity						
White	Reference		Reference		Reference	
Latino/Hispanic	−172.5 (−456.1; 111.1)	0.220	−126.5 (−407.3; 154.3)	0.360	−331.5 (−735.1; 72.1)	0.102
Black	**328.8 (39.0; 618.6)**	**0.028**	**444.7 (150.7; 738.7)**	**0.005**	−139.3 (−561.4; 282.8)	0.500
Asian	−31.9 (−460.4; 396.7)	0.879	71.9 (−442.4; 586.1)	0.774	−307.7 (−740.6; 125.1)	0.154
Native American	56.2 (−160.2; 272.6)	0.595	177.3 (−53.7; 408.2)	0.125	**−385.6 (−770.1; −1.2)**	**0.049**
Other	46.9 (−773.4; 867.3)	0.906	19.7 (−796.9; 836.3)	0.960	303.7 (−1115.2; 1722.6)	0.661
Sexual minority						
No	Reference		Reference		Reference	
Yes	**−676.4 (−1223.6; −129.2)**	**0.018**	**−616.7 (−1185.7; −47.6)**	**0.035**	**908.7 (−1524.9; −292.5)**	**0.006**
Maybe	**−958.3 (−1410.3; −506.3)**	**<0.001**	**−946.2 (−1473.5; −419.0)**	**0.001**	**−852.9 (−1366.1; −339.7)**	**0.002**
Don’t understand the question	−21.7 (−557.2; 513.8)	0.934	−272.0 (−805.7; 261.7)	0.301	619.4 (−87.5; 1326.2)	0.083
Decline to answer	−334.6 (−1411.3; 742.1)	0.525	−57.7 (−1301.3; 1186.0)	0.924	**−1044.0 (−1935.0; −153.0)**	**0.024**
Household income						
≤$24,999	Reference		Reference		Reference	
$25,000–$49,999	−264.4 (−713.1; 184.3)	0.234	−279.6 (−670.8; 111.5)	0.152	−246.0 (−879.6; 387.5)	0.428
$50,000–$74,999	213.3 (−302.9; 729.5)	0.400	210.9 (−265.2; 687.0)	0.367	289.1 (−418.4; 996.6)	0.405
$75,000–$99,999	254.4 (−158.9; 667.8)	0.214	214.5 (−198.6; 627.6)	0.292	412.8 (−101.8; 927.4)	0.110
$100,000–$199,999	**432.2 (14.2; 850.2)**	**0.043**	**406.3 (20.5; 792.1)**	**0.040**	467.4 (−146.6; 1081.4)	0.128
≥$200,000	**1003.3 (630.3; 1376.3)**	**<0.001**	**944.8 (555.3; 1334.3)**	**<0.001**	**1189.1 (641.5; 1736.7)**	**<0.001**
Average years of parent education	24.6 (−25.9; 75.0)	0.323	26.6 (−17.6; 71.0)	0.224	18.8 (−56.6; 94.1)	0.610
Parent’s marital status						
Parent married/partnered	Reference		Reference		Reference	
Parent not married/unpartnered	**−326.3 (−524.6; −128.0)**	**0.003**	**−266.3 (−494.8; −37.9)**	**0.024**	**−522.2 (−754.2; −290.1)**	**<0.001**
Data collection period						
Pre-COVID	Reference		Reference		Reference	
Peri-COVID	**−2133.5 (−2541.2; −1725.7)**	**<0.001**	**−2656.0 (−3157.0; −2155.1)**	**<0.001**	**−578.3 (−952.4; −204.2)**	**0.004**

Bold indicates *p* < 0.05. *B* coefficient from mixed-effect model. Models represent the abbreviated output from three mixed-effect models, including adjustment for age, sex, race/ethnicity, sexual orientation, household income, parent education, parent marital status, data collection period, and study site in addition to all variables listed. Propensity weights from the Adolescent Brain Cognitive Development Study were applied based on the American Community Survey from the US Census.

We conducted exploratory linear regression analyses examining sociodemographic associations with steps d^−1^ stratified by income, given evidence of significant effect modification by household income for Black adolescents in post hoc analyses (*p* for Black race × household income interaction = 0.004). There were some notable differences by race/ethnicity and household income level. For Black adolescents compared to White adolescents, those in high-income households recorded 523.4 (95% CI, −12.8; 1059.5) fewer steps d^−1^; however, in low-income households, Black compared to White adolescents recorded 135.9 (95% CI, −264.5; 536.3) more mean steps d^−1^ (*p* = 0.055 and *p* = 0.488, respectively). We did not find evidence of effect modification by income for other races/ethnicities.

We also conducted exploratory linear regression analyses examining sociodemographic associations with steps d^−1^ stratified by sex in response to post-hoc analyses finding effect modification by sex for Asian adolescents (*p* for Asian race × sex interaction=0.003). Asian male adolescents recorded 344.6 (95% CI, −111.3; 800.7) fewer steps d^−1^ compared to White male adolescents; however, this difference was not statistically significant (*p* = 0.131). In contrast, Asian female adolescents recorded 671.1 (95% CI, 85.2 to 1257.1) more steps d^−1^ compared to White female adolescents (*p* = 0.027).

## Discussion

In this population-based, demographically diverse sample of 10-to-14-year-old children in the U.S., we identified several notable sociodemographic factors associated with total mean steps d^−1^ prior to and during the COVID-19 pandemic. We found that older age was associated with fewer steps d^−1^, male sex was associated with significantly more steps d^−1^ than female sex, and sexual minority and questioning adolescents had fewer steps d^−1^ than heterosexual adolescents. Additionally, adolescents from lower-income households or those having unpartnered parents generally demonstrated fewer steps d^−1^. As expected, the COVID-19 pandemic was associated with markedly fewer steps d^−1^ across all categories, potentially attributed to the cancellation of schools, physical education classes, and sports teams, as well as closures of recreation and gym facilities.^2,27,28^ Black race was associated with more steps d^−1^ than White race overall, although this association was modified when household income was considered.

Our study confirmed findings from previous self-report studies regarding sex disparities in physical activity.^13^ Male sex was associated with significantly higher step count than female sex. Studies have shown that various factors, including weaker social influences, lead to lower physical activity among females.^29^ Female participation in physical education and school sports significantly declines in the transition from childhood to adolescence, potentially due to negative experiences in and perceptions of school-based physical education.^30^ This decline in participation in school sports may even extend to being less active on the weekends, when games or practices typically occur. Our study’s confirmation of these findings with device-based measurements rather than self-reported data highlights the need for interventions at the social and policy level to target this sex gap in physical activity.

In previous self-report-based studies, White race and higher income were associated with greater physical activity; thus, we hypothesized that Black adolescents included in our study would have lower step counts per day than their White counterparts. Contrary to our expectation, we found that Black race was associated with higher step counts overall. When utilizing self-report-based measures of leisure-time physical activity, it was observed that Black adolescents were typically less active than White adolescents.^13^ However, device-based measures provide an estimate of accumulated daily physical activity, which includes more than just the leisure-time domain (i.e., active transport, occupational, and household activities). Structural, social, and economic factors, including transportation infrastructure, neighborhood distributions, or other systemic inequities, may explain race differences, consistent with the biopsychosocial model and social epidemiology frameworks.^10–12^ For example, a larger proportion of the Black population reside in urban areas when compared to White populations. This may lead to greater reliance on walking as a mode of transportation among adolescents.^31^ Furthermore, Black people are more likely to be living in geographically and economically isolated communities compared to White people, which can result in greater distances to essential services and institutions, such as schools, parks, libraries, etc.^32^ Additionally, Black families are less likely to own an automobile.^33^ Therefore, Black adolescents may rely more heavily on public transportation or walking compared to White adolescents. While a higher step count as a measure of physical activity is associated with better health outcomes, the pattern we identified in this study could likely be based on systemic inequities tied to neighborhood, race, and class. An interesting point of consideration for future research is whether the positive health effects of an increased step count can be overshadowed by systemic inequities like environmental pollution and healthcare accessibility.

It is also important to note how these findings were modified by income level. Among participants from high-income households, Black race was associated with fewer steps than White race, which was the opposite of what we observed when looking at the association among low-income households. These findings may be explained by the Marginalization-Related Diminished Returns theory,^25^ which proposes that unlike other minorities or marginalized groups, even when socioeconomic resources are taken into consideration, Black individuals continue to experience health disparities.^25^ These diminished returns are attributed to the societal and systemic barriers that are created and facilitated by structural racism. Among low-income adolescents, Black race is associated with higher step count, which could likely be due to the systemic transportation inequities mentioned above. Conversely, among high-income adolescents, Black race is associated with lower step count, likely due to persistent societal barriers such as unequal access to parks, sports programs/facilities, or racism and discrimination.^2,34^ Black adolescents from high-income families have reported higher odds of perceived racism than Black adolescents from low-income families,^34,35^ which could partially explain the observed differences in step count. For instance, Black adolescents could be dissuaded from team sports participation in high-income contexts, where schools might benefit from diversity, equity, and inclusion curricula. Nevertheless, given the exploratory nature of these findings, more research is needed to better understand the modification of outcomes by income level and other social and structural factors among Black adolescents.

In addition, our study showed that Native American adolescents^16^ had a lower weekend step count than White adolescents, which is consistent with previous studies that have shown Native American adolescents to be less active than non-Native American peers.^2,36^ These same differences were not observed during the weekdays, when school attendance, physical education classes, or sports practice may equalize step count. This finding may be partially attributed to rural neighborhood environments with less access to public parks, open space, and private recreation facilities.^36^

Sexual minority (e.g., lesbian, gay, bisexual) adolescents recorded fewer steps compared to their heterosexual peers. A prior study of adolescents and young adults 12–22 years similarly found that sexual minorities self-reported less moderate-to-vigorous intensity physical activity and less participation in team sports compared to their heterosexual peers.^37^ We build on these previous findings by focusing on early adolescents and incorporating device-based data on physical activity, which is less susceptible to response or recall bias. Potential reasons for lower physical activity among sexual minority adolescents previously investigated include lower athletic self-esteem and gender nonconformity.^37^ Notably, participants responding “maybe” to the sexual minority question recorded fewer steps compared to their heterosexual peers, suggesting that individuals who are uncertain about their sexual orientation share similar step patterns as sexual minorities, potentially due to sharing similar psychological and social stressors. Some of the participants responding “maybe” to the sexual minority question could identify as sexual minorities later in life. The average age for sexual orientation identification among adolescents is 17.8 years.^38^

The limitations of this study should be noted. Fitbit device data were collected for a 3-week period (21 days), which is longer than the typical seven-day protocol in prior studies.^8^ However, this may not be representative of a participant’s physical activity over the course of one year. Future studies could consider pre-registering and utilizing longitudinal Fitbit data for longer durations to overcome this limitation and explore potential seasonal disparities. Additionally, step count alone does not account for the intensity of physical activity. While only days with >599 min of waking wear were included in the analysis, variations in wear time above this threshold were not controlled for, thus producing a potential limitation with regards to differences in participants’ wake versus sleep time or potentially not wearing the Fitbit during some sports or activities due to uniform needs or discomfort. Another limitation is the potential for unmeasured confounders, although we did control for site and sociodemographic factors as well as data collection period. Furthermore, Fitbit may overestimate number of steps d^−1^, which is a limitation to consider for total step counts among adolescents in the study.^39–41^ The strengths of this study include the large, sociodemographically diverse population-based sample and the use of device-based data that are not limited by biases associated with self-reported data.

Given the sociodemographic differences in adolescent physical activity, as measured by steps d^−1^ across a racially/ethnically diverse population-based sample, physical activity guidelines and interventions should target key populations and adapt strategies to accommodate diverse backgrounds. To inform these guidelines, steps can serve as an easily understandable and objectively measured physical activity guideline target. Interventions focusing on early adolescents are especially important, as this age range may establish future patterns of activity that can be continued through adolescence and into adulthood. For example, targeted physical education programs and social support for female adolescents may be considered to encourage physical activity participation in this population.^42,43^ Communities may also prioritize interventions to increase physical activity accessibility in low-income neighborhoods, including safe outdoor recreation spaces and after-school sports/extracurricular participation support programs.^44^ Future studies could aim to utilize wearable data to gain deeper insights into the root causes of the disparities we observed, especially the lower step count amongst female adolescents and the racial disparity patterns among low- versus high-income households.

## Data Availability

Data used in the preparation of this article were obtained from the ABCD Study (https://abcdstudy.org), held in the NIMH Data Archive (NDA). Investigators can apply for data access through the NDA (https://nda.nih.gov/).
